# Increasing CD44^+^/CD24^- ^tumor stem cells, and upregulation of COX-2 and HDAC6, as major functions of HER2 in breast tumorigenesis

**DOI:** 10.1186/1476-4598-9-288

**Published:** 2010-11-02

**Authors:** Kai-Hung Wang, An-Pei Kao, Chia-Cheng Chang, Jau-Nan Lee, Ming-Feng Hou, Cheng-Yu Long, Hung-Sheng Chen, Eing-Mei Tsai

**Affiliations:** 1 Graduate Institute of Medicine, Kaohsiung Medical University, Kaohsiung, Taiwan; 2 Center of Excellence for Environmental Medicine, Kaohsiung Medical University, Kaohsiung, Taiwan; 3 Department of Obstetrics and Gynecology, Kaohsiung Medical University Hospital, Kaohsiung Medical University, Kaohsiung, Taiwan; 4 Division of General & Gastroenterological Surgery, Department of Surgery, Kaohsiung Medical University Hospital, Kaohsiung Medical University, Kaohsiung, Taiwan; 5 National Sun Yat-Sen University-Kaohsiung Medical University Joint Research Center, Kaohsiung, Kaohsiung, Taiwan; 6 Cancer Center, Kaohsiung Medical University Hospital, Kaohsiung Medical University, Kaohsiung, Taiwan; 7 Department of Obstetrics and Gynecology, Kaohsiung Municipal Hsiao-Kang Hospital, Kaohsiung, Taiwan; 8 Department of Pediatrics and Human Development, Michigan State University, East Lansing, Michigan, USA

## Abstract

**Background:**

Cancer cells are believed to arise primarily from stem cells. CD44^+^/CD24^- ^have been identified as markers for human breast cancer stem cells. Although, HER2 is a well known breast cancer oncogene, the mechanisms of action of this gene are not completely understood. Previously, we have derived immortal (M13SV1), weakly tumorigenic (M13SV1R2) and highly tumorigenic (M13SV1R2N1) cell lines from a breast epithelial cell type with stem cell phenotypes after successive SV40 large T-antigen transfection, X-ray irradiation and ectopic expression of HER2/C-erbB2/neu. Recently, we found that M13SV1R2 cells became non-tumorigenic after growing in a growth factor/hormone-deprived medium (R2d cells).

**Results:**

In this study, we developed M13SV1R2N1 under the same growth factor/hormone-deprived condition (R2N1d cells). This provides an opportunity to analyze HER2 effect on gene expression associated with tumorigenesis by comparative study of R2d and R2N1d cells with homogeneous genetic background except HER2 expression. The results reveal distinct characters of R2N1d cells that can be ascribed to HER2: 1) development of fast-growing tumors; 2) high frequency of CD44^+^/CD24^- ^cells (~50% for R2N1d vs. ~10% for R2d); 3) enhanced expression of COX-2, HDAC6 mediated, respectively, by MAPK and PI3K/Akt pathways, and many genes associated with inflammation, metastasis, and angiogenesis. Furthermore, HER2 expression can be down regulated in non-adhering R2N1d cells. These cells showed longer latent period and lower rate of tumor development compared with adhering cells.

**Conclusions:**

HER2 may induce breast cancer by increasing the frequency of tumor stem cells and upregulating the expression of COX-2 and HDAC6 that play pivotal roles in tumor progression.

## Background

Breast cancers and other cancers are believed to arise primarily from stem cells [1] through a series of genetic and epigenetic alterations facilitated by mechanisms of tumor initiation, promotion and genomic instability [2]. One of the best known breast cancer oncogenes is HER2 (also known as neu, ErbB-2, and NGL) which belongs to the epidermal growth factor receptor (EGFR) family [3,4] and encodes an 185 kDa transmembrane receptor tyrosine-kinase [5-7]. Human HER2 oncogene and its p185^HER2/neu ^oncoprotein are overexpressed in 20-30% of invasive breast cancers [8,9] and have been associated with cytotoxic and endocrine drug therapy resistance [10]. The mechanisms of action of HER2 over-expression that cause tumor development and enhance the intrinsic metastatic potential of breast cancer [11] are not completely understood. However, few major mechanisms have been reported. First, HER2 could regulate cyclooxygenase (COX)-2 [12] and elevated COX-2 could induce many tumorigenic effects such as tumor invasion, angiogenesis, suppression of host immunity, resistance to apoptosis [13-15] and epithelial to mesenchymal transition (EMT) [16]. Second, p185^HER2/neu ^could phosphorylate and activate major signalling pathways such as phosphatidylinositol-3-kinase (PI3K/Akt) and mitogen-activated protein kinase (MAPK) pathways and promote cell survival, tumor growth and metastasis [10,17,18]. Conversely, anti-HER2 antibody, Herceptin, could inhibit PI3K/Akt and result in up-regulation of p27, down-regulation of cyclin D1 and antitumor action [19]. Thirdly, HER2 has been reported to increase the size and frequency of mammospheres that contain breast epithelial progenitor cells and to expand normal mammary epithelial cells that express the stem cell marker, aldehyde dehydrogenase (ALDH) [20]. Furthermore, ectopic expression of HER2 in human mammary carcinoma cells could increase ALDH-positive cells, indicating that HER2 could enhance the frequency of both normal and cancer stem cells [20].

We have previously reported the isolation of a human breast epithelial cell type (Type-1 HBEC) from reduction mammoplasty of healthy women with stem cell characteristics [21]. These cells are characterized by deficiency in gap-junctional intercellular communication [21], the ability to form budding and ductal organoids on Matrigel [22], the expression of luminal epithelial cell markers (i.e. epithelial membrane antigen and cytokeratin 18) [21], estrogen receptor-alpha (ERα) [23] and the stem cell pluripotency gene Oct-4 [24], similar to the phenotypes of breast carcinoma cells such as the MCF-7 cell line. Furthermore, Type-1 HBECs were highly susceptible to telomerase activation and immortalization following SV40 large T-antigen transfection [25] which is known to inactivate p53 and Rb as well as to transactivate a CCAAT box binding factor (CBF/cdc2) [26,27]. Both changes have been reported for human breast cancer. These immortal cells (M13SV1) can be further transformed to weakly tumorigenic (M13SV1R2) and highly tumorigenic cells (M13SV1R2N1) by successive X-ray irradiation and ectopic expression of C-erbB2/neu [28]. Recently, we found that M13SV1R2 cells became non-tumorigenic after growing in a growth factor/hormone-deprived medium for >10 passages (referred to as R2d cells) [16]. Unlike M13SV1R2 cells, these R2d cells contain CD44^+^/CD24^- ^cells previously identified as breast cancer stem cells [29] and were responsive to estrogen for cell growth and tumor development [16]. In this study, we developed M13SV1R2N1 under the same growth factor/hormone-deprived condition (referred to as R2N1d cells). This provides an opportunity to analyze unambiguously the effects of HER2 on tumor development and gene expressions underlying tumorigenic mechanisms by comparative study of R2d and R2N1d cells with homogeneous genetic background under same cell culture condition.

## Results

### Development of R2N1d cells

**Figure 1:**
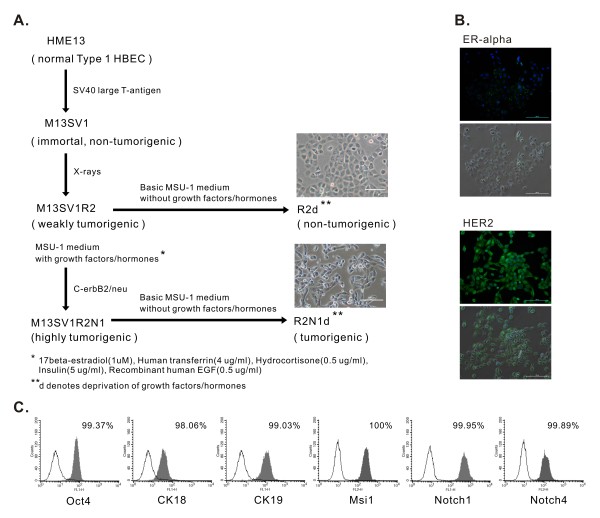
**Derivation of a tumorigenic cell line (R2N1d) and characteristics of R2N1d cells**. **A**, a diagram showing the development of R2d cells which are non-tumorigenic and R2N1d cells which are highly tumorigenic. Both R2d and R2N1d cells were derived from the same immortal cell line M13SV1 and cultured for >10 passages under same cell culture condition before experiment using MSU-1 medium without growth factors and hormones (except 5% FBS). **B**, expression of ER-α and HER2 in R2N1d cells by immunocytochemical staining (green fluorescence). Cell nuclei were stained with DAPI and recognized as blue fluorescence (top figure, image observed under fluorescence microscope; bottom figure, the mergence of fluorescence and phase images, scale bar = 100 μm). **C**, R2N1d cells were labelled with antibodies against CK-18, CK-19, Msi1, Notch-1, Notch-4 and Oct-4 for immunophenotyping by flow cytometric analysis. The open histograms indicate background signal and shaded histograms showing positive reactivity.

In order to investigate HER2 effect on tumor development and gene expression, M13SV1R2N1 cells were cultured under similar condition as R2d cells, i.e. in MSU-1 medium [21] without growth factors/hormones except 5% FBS for more than 10 passages, (referred to as R2N1d) (Figure 1A). Morphologically, R2N1d cells were more heterogeneous, i.e. increased intercellular separation and scattering of cells and the formation of pseudopodia (Figure 1A). Similar to parental M13SV1R2N1 cells, R2N1d cells expressed ERα and HER2 by immunocytochemical study (Figure 1B).

### R2N1d cells expressed stem cell-related genes

The Notch signaling pathway is implicated in the regulation of cell differentiation and self-renewal of mammary stem cells. Over-expression of the active form of Notch 4 inhibits differentiation of breast epithelial cells [30]. Musashi-1 (Msi-1) is a positive regulator of Notch signaling, and both Msi-1 and Notch 1 are key regulators of asymmetrical cell division in human breast epithelial stem cells [31]. We have examined whether R2N1d cells express genes involved in stem cell function and self-renewal, i.e. Oct-4 [24] and Notch pathway [32] by flow cytometric analysis. The results show that R2N1d cells expressed the stem cell pluripotency gene, Oct-4, (99.37%) and the Notch pathway-related genes, Notch1 (99.95%), Notch4 (99.89%) and Msi1 (100%) (Figure 1C). The expression of these genes in R2N1d was similar to R2d cells (data not shown). Similar to parental normal Type 1 HBECs [21,22,24] and immortal R2d [16] cells, as well as human breast carcinoma cells such as MCF-7, the R2N1d cells expressed Oct-4 and luminal epithelial cells markers, cytokeratin 18 and 19 (Figure 1C).

### Comparison of gene expression profiles between R2d cells and R2N1d cells

**Figure 2:**
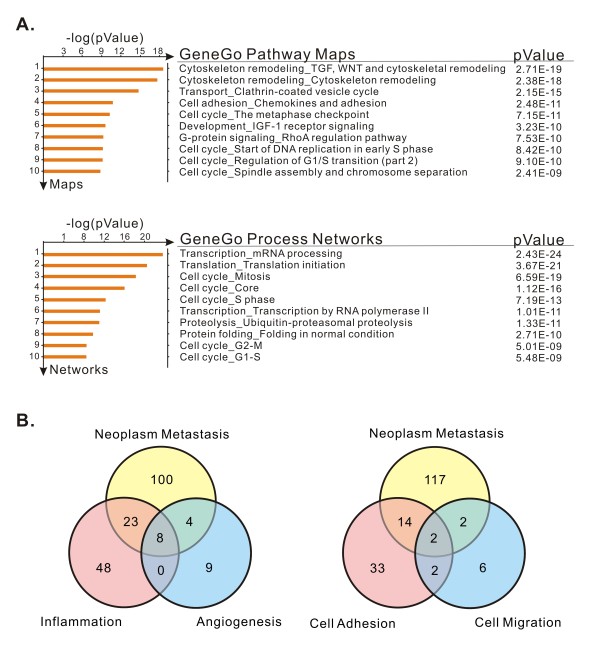
**Functional analysis of genes significantly up-regulated in R2N1d cells compared with R2d cells**. **A**, genes involved in top ten of GeneGo pathway maps and top ten GeneGo process networks by MetaCore analysis. **B**, number of up-regulated genes belongs to 5 functional categories. Eight genes are at once correlated with metastasis, inflammation and angiogenesis, i.e. *TNFRSF12A*, *CEACAM1*, *PLAU*, *HIF1A*, *IL8*, *HMOX1*, *VEGFC *and *IL1B*, whereas two genes are correlated with adhesion, metastasis, and migration, i.e. *CD44 *and *LAMC1*.

R2d and R2N1d cells were derived from the same parental cell. Their cellular contexts are presumably similar except the integration and expression of the HER2/neu gene in R2N1d cells. In order to examine the mechanism of action of HER2 in human breast tumor development, we analyzed the differential gene expression profiles between R2d and R2N1d cells, using the HumanWG-6 BeadChip. The mRNA expression of R2d and R2N1d cells were compared in a scatter plot. They presented similar patterns in Pearson correlation R^2 ^of 0.7821. Out of the genes screened, 3289 genes in R2N1d cells were found to be upregulated by more than 5-folds in comparison with R2d cells, while none was found to be down-regulated by more than 5-folds. Further analysis of the total genes by MetaCore reveals high expression of genes involved in cytoskeleton remodeling, cell adhesion and cell cycle progression in the top ten GeneGo pathway maps (Figure 2A). There was elevated expression of genes related to transcription, translation and cell cycle processes among the top ten GeneGo process networks (Figure 2A). For investigation of HER2 function in R2N1d cells, we focused on gene expression related to cell adhesion, metastasis, inflammation, angiogenesis and migration. In these categories of function, many genes were elevated in R2N1d cells: 51, 135, 79, 21 and 12 genes, respectively, for cell adhesion, metastasis, inflammation, angiogenesis and migration (see Additional file 1, Table S1). There are eight genes that are at once correlated with metastasis, inflammation and angiogenesis, i.e. *TNFRSF12A*, *CEACAM1*, *PLAU*, *HIF1A*, *IL8*, *HMOX1*, *VEGFC *and *IL1B*. Two genes are simultaneously correlated with adhesion, metastasis, and migration, i.e. *CD44 *and *LAMC1 *(Figure 2B). The altered expression of some selected genes was subsequently confirmed by q-PCR. The HER2 overexpression in R2N1d cells was also detected in this study (Table 1).

**Table 1:** Comparison of fold increase of RNA transcript between R2N1d and R2d cells by qPCR analysis

Gene Name	Symbol	Description	Up-/Down-Regulation
AACT/ACT	SERPINA3	Serpin peptidase inhibitor, clade A (alpha-1 antiproteinase, antitrypsin), member 3	12.50
HER2/HER2	ERBB2	V-erb-b2 erythroblastic leukemia viral oncogene homolog 2, neuro/glioblastoma derived oncogene homolog (avian)	6.38
ASBABP2/DIPLA1	PAPPA	Pregnancy-associated plasma protein A, pappalysin 1	3.91
AIS/DHTR	AR	Androgen receptor (dihydrotestosterone receptor; testicular feminization; spinal and bulbar muscular atrophy; Kennedy disease)	3.50
TSP2	THBS2	Thrombospondin 2	3.30
CORNIFIN/GADD33	SPRR1B	Small proline-rich protein 1B (cornifin)	3.14
COX-2/COX2	PTGS2	Prostaglandin-endoperoxide synthase 2 (prostaglandin G/H synthase and cyclooxygenase)	2.59
IBP2/IGF-BP53	IGFBP2	Insulin-like growth factor binding protein 2, 36kDa	2.49
CD271/Gp80-LNGFR	NGFR	Nerve growth factor receptor (TNFR superfamily, member 16)	2.41
Cyclin A1	CCNA1	Cyclin A1	2.36
ARMD9/ASP	C3	Complement component 3	2.22
BCEI/D21S21	TFF1	Trefoil factor 1	2.13
STC-2/STCRP	STC2	Stanniocalcin 2	-2.21
BAG-1	BAG1	BCL2-associated athanogene	-2.34
GIG8/ID2A	ID2	Inhibitor of DNA binding 2, dominant negative helix-loop-helix protein	-2.48
DKFZp686A04236/HMG1	HMGB1	High-mobility group box 1	-2.54
CD49f/ITGA6B	ITGA6	Integrin, alpha 6	-2.56
DKFZp686N23123/ER	ESR1	Estrogen receptor 1	-2.64
Beta-NGF/HSAN5	NGF	Nerve growth factor (beta polypeptide)	-2.68
GIF/GIFB	MT3	Metallothionein 3	-3.88
CBF-A/CBF-B	NFYB	Nuclear transcription factor Y, beta	-4.04

### HER2 overexpression enhanced cyclooxygenase-2 expression through MAPK pathway

**Figure 3:**
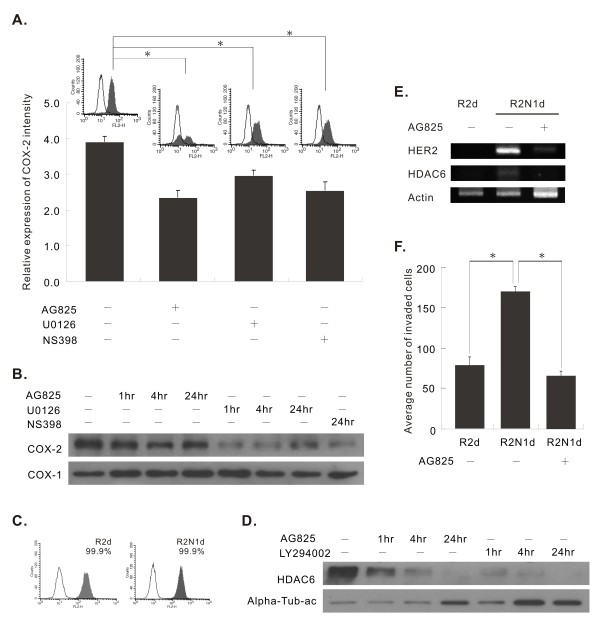
**HER2 effects on COX-2 and HDAC6 expression and cell invasion**. **A**, the effect of AG825 (a HER2 tyrosine kinase inhibitor, 25 μM), U0126 (a highly selective inhibitor of MAPK/ERK kinase, 10 μM) or NS398 (a COX-2 inhibitor, 100 μM) treatment on COX-2 protein expression in R2N1d cells by flow cytometry analysis. **B**, the effect of AG825, U0126 or NS398 treatment on COX-1 and COX-2 expression in R2N1d cells by western blotting analysis. **C**, the expression of HDAC6 in R2d and R2N1d cells by flow cytometry analysis. **D**, the effect of AG825 (25 μM) and LY294002 (10 μM) (a PI3K inhibitor) treatment on HDAC6 expression in R2N1d cells by western blotting analysis. **E**, the effect of AG825 (25 μM) treatment for 24 hr on HER2 and HDAC6 expression in R2d and R2N1d cells by RT-PCR analysis. F, the effect of HER2 inhibitor, AG825 (25 μM), treatment for 24 hr on invasion ability of R2N1d cells, assayed by using the invasion chamber. R2d cells were also included in the experiment.

In literature, COX-2 overexpression is found in many different human cancers including breast cancer [33]. Overexpression of HER2 was associated with increased level of COX-2 [34]. Therefore, we carried out experiments to determine if HER2 could enhance the expression of COX-2 in R2N1d cells. By qPCR analysis, the mRNA level of COX-2 was, indeed, significantly increased in R2N1d cells (2.6-folds) compared to R2d cells (Table 1). The HER2 effect on up-regulation of COX2 expression is confirmed by western blot analysis as shown in Figure 3B (R2N1d, lane 1 and R2N1d treated with AG825, lane 4) or when R2N1d (Figure 3B, lane 1) and R2d cells (Figure 3A, lane 1 in reference 16) are compared (Additional file 2, Figure S1). Furthermore, by flow cytometric analysis, the treatment with a selective ATP-competitive inhibitor of the tyrosine kinase activity of HER2 (AG825, 25 μM) partially negated the expression of COX-2. A highly selective inhibitor of MAPK/ERK (U0126, 10 μM) and a selective inhibitor of COX-2 (NS398, 100 μM) also partially negated the expression of COX-2 (Figure 3A). This HER2 mediated COX-2 expression through MAPK pathway was confirmed by western blotting. As shown in Figure 3B, AG825 and U0126 caused a marked decrease in COX-2 expression in a time-dependent manner in R2N1d cells; as expected, NS398 treatment blocked the expression of COX-2 protein. In contrast, all these 3 inhibitors did not modulate the expression of COX-1 (Figure 3B). Overall, these experiments provide evidence that HER2 could up-regulate COX-2 expression through MAPK pathway in R2N1d cells.

### HDAC6 as a HER2-regulated gene in R2N1d cells

Histone deacetylases (HDACs) have been linked to pathogenesis of cancer [35]. Among them, HDAC6 has been shown to be required for efficient oncogenic transformation and tumor formation [36,37]. HDAC6 could regulate cytoskeleton, cell adhesion, cell motility and migration [37-39]. A study was carried out to determine if HER2 could affect the expression of HDAC6 in R2N1d cells which express the HDAC6 by flow cytometric analysis (99.85%) (Figure 3C). By western blotting analysis, we detected a decrease of HDAC6 protein after 24 h treatment with the HER2 inhibitor, AG825, indicating that the expression of HDAC6 is regulated by HER2 in R2N1d cells. The relative amount of acetylated alpha-tubulin was found to be inversely correlated with the expression of HDAC6 (Figure 3D). The effect of HER2 on HDAC6 expression was also revealed by the higher expression of this gene in R2N1d compared with R2d cells (2.41 fold) (last listing in Additional file 1, Table S1) and by RT-PCR analysis (Figure 3E).

### PI3K inhibitor, LY294002, repressed the expression of HDAC6 in R2N1d cells

HDAC6 could contribute to tumorigenesis by facilitating the activation of PI3K/Akt pathway [34]. It is, however, not clear if the expression of HDAC6 could be affected by PI3K activation. By western blotting analysis, we detected a decrease of HDAC6 protein expression in R2N1d cells after 24 h treatment with the PI3K inhibitor, LY294002 (10 μM), while relative amount of acetylated alpha-tubulin was inversely correlated with the expression of HDAC6 (Figure 3D). The results indicate that HDAC6 expression could be regulated by PI3K/Akt activity.

### HER2 overexpression increased invasiveness

We have compared the invasion ability of R2d cells, R2N1d cells and R2N1d cells treated with HER2 inhibitor, AG825. The results reveal that R2N1d cells had a 2.2-fold higher invasion ability compared to R2d cells and R2N1d cells treated with AG825 cells had a 2.5-fold lower invasion ability compared to R2N1d cells without AG825 treatment (Figure 3F), indicating that HER2 enhanced the cell invasion ability.

### HER2 converted non-tumorigenic R2d cells into highly tumorigenic R2N1d cells

**Figure 4:**
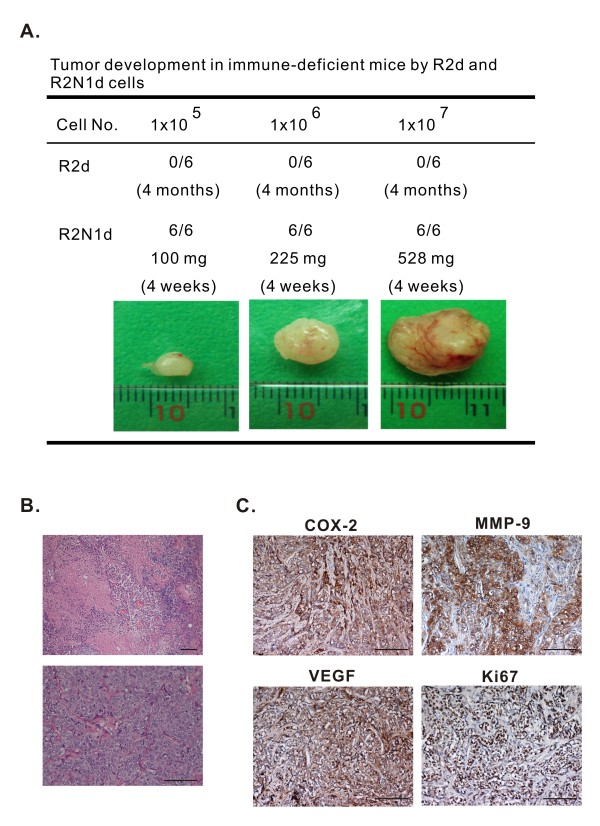
**Tumorigenicity of R2d and R2N1d cells, and genes expressed by tumors developed by R2N1d cells**. **A**, the development of tumors in immune-deficient mice after inoculation with different numbers of R2d or R2N1d cells (1 × 10^5^, 1 × 10^6 ^or 1 × 10^7 ^cells per site) for 4 months (R2d) or 4 weeks (R2N1d). The average size of tumors for each treatment are also determined. **B**, histological sections of resected tumor revealed sheets of polygonal cells with high nucleus/cytoplasm (N/C) ratio. **C**, these tumor cells expressed Ki67 (90%), VEGF (score 4), COX-2 (score 4) and MMP-9 (score 4). The length of the scale bar of these photos is 100 μm.

In our tumorigenesis study, different numbers of R2d or R2N1d cells (1 × 10^5^, 1 × 10^6 ^or 1 × 10^7^) were subcutaneously injected into immune-deficient mice for tumor development. Those mice inoculated with low or high number (1 × 10^5 ^or 1 × 10^7 ^cells) of R2d cells failed to develop tumor 24 weeks after inoculation (Figure 4A). In contrast, R2N1d cells, developed visible and palpable tumors in 2 weeks in mice inoculated with as low as 1 × 10^5 ^cells. Tumors developed from different numbers of R2N1d cells were harvested at 4 weeks after inoculation for quantitative measurement of tumors and pathological analysis. The results show that tumor sizes were closely dependent on numbers of R2N1d cells initially inoculated, i.e. 100 ± 18 mg, 225 ± 36 mg, 528 ± 82 mg, respectively for 1 × 10^5^, 1 × 10^6 ^, 1 × 10^7 ^cells inoculated (Figure 4A). These tumors were processed for immunohistochemical study. Histological sections of the resected tumor revealed sheets of cells with nuclear enlargement, high nucleocytoplasmic (N/C) ratio, hyperchromasia and pleiomorphism. There were areas where tumor cells invaded neighboring tissue (Figure 4B). These tumor cells showed the expression of Ki-67 (90%), VEGF (score 4), COX-2 (score 4) and MMP-9 (score 4) that are known to be expressed in invasive and metastatic tumors (Figure 4C). These results clearly show that R2N1d cells with HER2 overexpression were highly tumorigenic, whereas R2d cells were non-tumorigenic.

### High frequency of R2N1d cells expressed breast cancer stem cell markers

**Figure 5:**
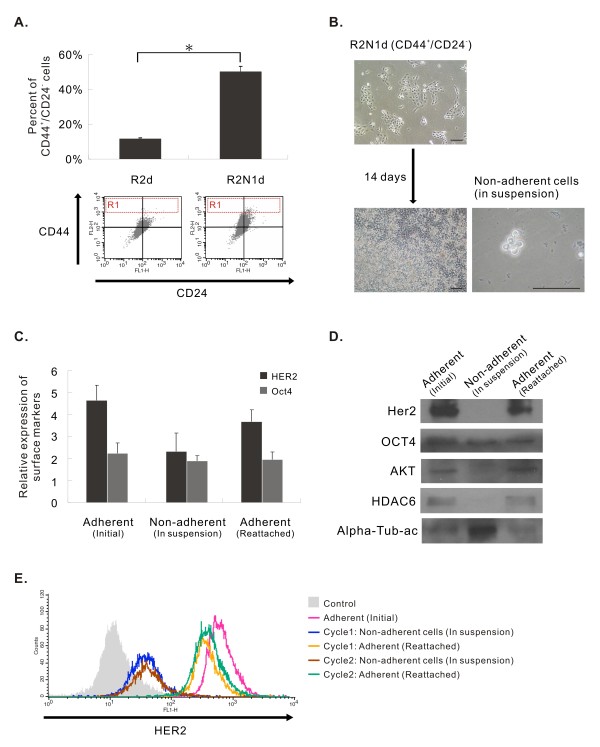
**Expression of breast cancer stem cell markers CD44^+^/CD24^-/low ^in R2d and R2N1d cells and Modulation of HER2, Oct-4, AKT and HDAC6 expression in R2N1 cells by cell culture condition**. **A**, higher frequency of CD44^+^/CD24^-/low ^cells was found in R2N1d cells than R2d cells (~50% vs. ~10%). R1 region denotes a small population of CD44^high ^cells which was increased in R2N1d cells. The fluorescence cut off level: high expression was fluorescence intensity > 10^3^; negative or low expression was fluorescence intensity < 10^2^. **B**, CD44^+^/CD24^- ^cells sorted by flow cytometry tend to show contact-insensitive growth in confluent culture and gave rise to non-adherent cells in suspension. By flow cytometric analysis (**C, E)**, and by western blotting (**D**), adherent cells and re-attached cells were found to be HER2^+^/OCT4^+^/AKT^+^/HDAC6^+^, whereas non-adherent cells were HER2^-^/OCT4^+^/AKT ^-^/HDAC6^-^. The length of the scale bar of these photos is 100 μm.

Since cancer stem cells initiate and sustain tumor growth, these cells are also considered as targets for cancer therapy. For breast cancer, CD44^+^/CD24^-/low ^[29] and aldehyde dehydrogenase (ALDH) [40] have been reported as markers for breast cancer stem cells. We examined the expression of CD44^+^/CD24^-/low ^in R2d and R2N1d cells by flow cytometric analysis. The results revealed a very high frequency of CD44^+^/CD24^-/low ^cells in R2N1d cells (50%) compared to that in R2d cells (10%) (Figure 5A). It is also noted that a subpopulation of CD44^high ^cells (Figure 5A, R1 region) appeared in R2N1d cell culture (1.5%). The results of this study clearly show that a major function of HER2 is to increase the frequency of CD44^+/high^/CD24^- ^cancer stem cells.

### Non-adherent R2N1d cells derived from adherent monolayer culture lost HER2 and CD44^+^/CD24^- ^expression

CD44^+^/CD24^- ^cells were sorted out from R2N1d cells by flow cytometry. These cells tend to show contact-insensitive growth (piling up) in monolayer and gave rise to non-adherent cells in suspension in extended growth (Figure 5B).

Experiments were carried out to compare gene expression of adherent and non-adherent R2N1d cells as well as reattached non-adherent cells after replating. By flow cytometric analysis and by western blotting, the results indicate that, while Oct-4 expressions were comparable in the 3 different populations of cells, the HER2 expression was significantly reduced in non-adherent cells (Figure 5C, D and 5E). After incubation of non-adherent R2N1d cells for 3 weeks, a few of these suspended cells re-attached and proliferated (colony-forming efficiency was 3/15000). These re-attached cells were found to express HER2 and Oct-4 similar to their parental adherent cells (Figure 5C, D and 5E).

Results presented previously (Figure 3D) show that HER2 and PI3K/Akt activity regulate the expression HDAC6. Consistent with this function, we found that adherent and reattached R2N1d cells expressed HER2 as well as AKT1 and HDAC6, whereas non-adherent R2N1d cells in suspension lost the expression of these 3 markers (Figure 5D). The expression of CD44^+^/CD24^- ^in non-adherent R2N1d cells was found to be dramatically reduced compared to adherent cells (Additional file 3, Figure S2), reaffirming the regulation of CD44^+^/CD24^- ^expression by HER2.

### HER2-negative R2N1d cells from suspension formed HER2 positive tumor at lower frequency and with longer latent period

**Figure 6:**
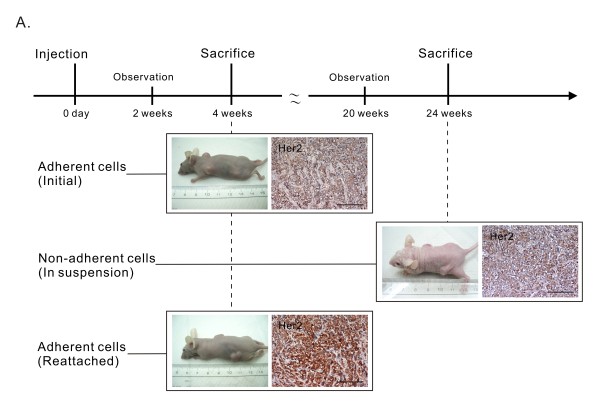
**Tumorigenicity of 3 types of R2N1d cells in immune-deficient mice**. Adherent (CD44^+^/CD24^- ^sorted), non-adherent and re-attached R2N1d cells were inoculated subcutaneously into nude mice for tumor development. Only those mice inoculated with adherent and reattached R2N1d cells showed visible and palpable tumors 4 weeks after inoculation; Mice inoculated with non-adherent R2N1d cells developed tumors after 24 weeks. Histological sections of resected tumor harvested at 4 weeks (from adherent and reattached cells) or 24 weeks (from non-adherent cells) showed HER2 expression. The length of the scale bar of these photos is 100 μm.

**Figure 7:**
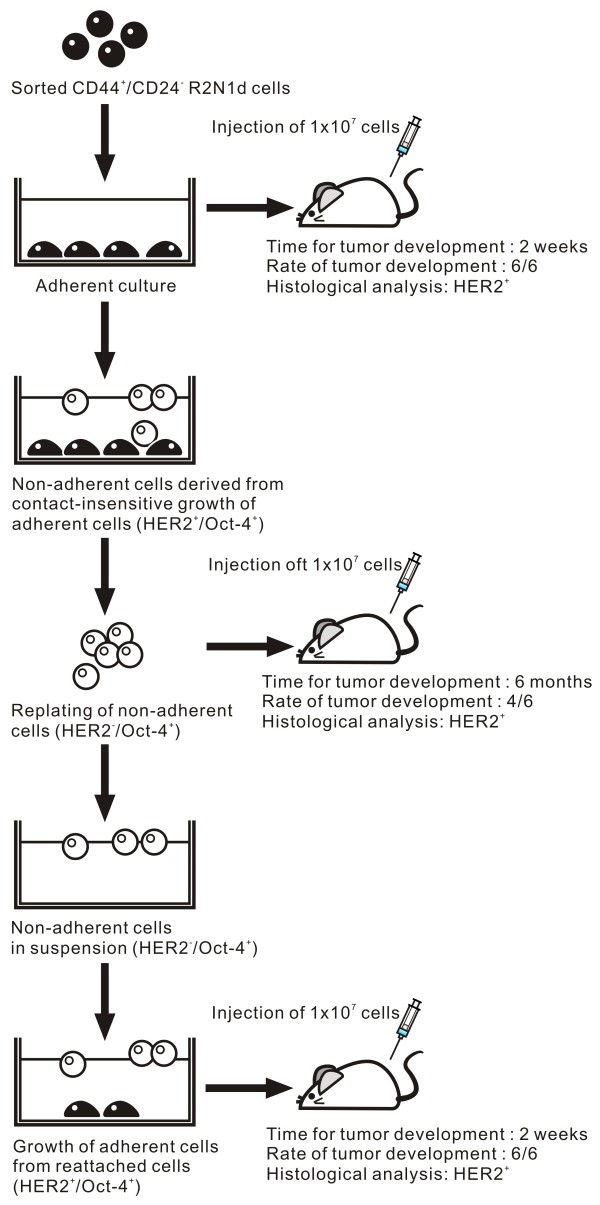
**A diagram depicting the change in HER2 expression and tumorigenicity of R2N1d (CD44^+^/CD24^- ^sorted) cells due to cell culture condition**. Adherent R2N1d cells were OCT4^+^/HER2^+ ^and highly tumorigenic (tumor developed in 1 month after inoculation), the non-adherent R2N1d cells detached from confluent R2N1d cells in suspension were OCT4^+^/HER2^- ^and took longer time (6 months) to develop tumor at lower frequency. The reattached R2N1d cells from non-adherent cells were similar to parental adherent cells in OCT4^+^/HER2^+ ^expression and tumor development.

Since we have the 3 different populations of R2N1d cells developed under different culture condition and with different expression of HER2 (i.e. adherent, non-adherent and reattached non-adherent cells), it is important to know if they differ in tumorigenicity in immune-deficient mice. The results show that both adherent and re-attached R2N1d cells developed tumors in 2 weeks with 1 × 10^7 ^cells. In contrast, the non-adherent R2N1d cells in cell suspension did not form palpable tumors until 6 months post-inoculation of the same number of cells (1 × 10^7 ^cells). The tumor-forming frequency of the non-adherent cells was also lower (4/6) compared with adherent and reattached cells (6/6), examined 6 months after subcutaneous injection of cells (Figure 6 and 7). All these newly formed tumors developed by the 3 types of cells expressed HER2 by immunohistochemical study (Figure 6A). These results reaffirmed the important role of HER2 in tumor development.

## Discussion and Conclusions

HER2 as an important breast cancer oncogene is well known from the frequent amplification or overexpression of this gene in aggressive breast tumors [8,9] and the efficacy of anti-HER2 antibody, Herceptin, in treatment of breast cancer with HER2 overexpression [41]. Although some functions and mechanisms of HER2 in breast tumor development have been delineated, the exact mechanisms of action of this gene have not been completely understood. By comparison of phenotypic differences of two cell lines derived from a common breast epithelial stem cell with homogeneous cellular context but differing in HER2 expression under same cell culture condition, we believe the results of this study should reveal more convincing and unambiguous information in regard to it's mechanism of function.

### The biological effects of HER2

The biological effects induced by HER2 as revealed by the phenotypic differences between R2N1d and R2d cells and from HER2 inhibitor study include 1) the morphological change from contact-sensitive R2d cell culture to contact-insensitive (piling up) R2N1d cells with increasing cell separation and motility (Figure 1A); 2) the development of fast-growing invasive tumors, in contrast to R2d cells which were non-tumorigenic (Figure 4A) [16], and; 3) increased cell invasion ability from HER2 inhibitor study (Figure 3F). Unlike R2dE (R2d developed in estrogen-containing medium) [16], the tumors developed by R2N1d cells did not require estrogen treatment and the resulting tumors were significantly larger. The average size of tumors developed by R2N1d cells (225 mg and 528 mg formed by 1 × 10^6 ^and 1 × 10^7 ^cells, respectively) is comparable to tumors formed by the parental M13SV1R2N1 cells which is 295 mg and 11.2 mm in diameter (formed by 6 × 10^6 ^cells) [28] and much larger than tumors developed by R2dE cells (3 mm in diameter) [16]. Other major phenotypes of R2d and R2N1d and their parental cell lines are summarized in Additional file 4, Table S2.

### Major effects of HER2 on gene expression

The comparison of gene expression profiles between R2d and R2N1d cells by using the Human WG-6 BeadChip reveals that many genes related to cell adhesion, migration, metastasis, inflammation and angiogenesis have been significantly activated (Table S1, Figure 2). The enhanced expression of these genes could be the primary effect of HER2 or secondary effect of few key genes induced by HER2. By using the micro-array analysis and other methods, i.e. qPCR, specific inhibitors, flow cytometric and western blotting analyses, few key genes with profound consequence in tumorigenesis have been found to be induced by HER2. These genes include COX-2, HDAC6 and breast cancer stem cell marker, CD44^+^/CD24^- ^.

COX-2 over-expression has been found in about 40% of cases of invasive breast carcinoma and at a higher frequency in preinvasive ductal carcinoma in situ (DCIS) [42]. By qPCR analysis, the level of COX-2 transcripts was significantly elevated in R2N1d cells (2.6 folds) compared with R2d cells (Table 1). The expression of COX-2 in R2N1d can be significantly decreased by treatment with HER2 inhibitor, AG825, or MAPK/ERK inhibitor, U0126, using flow cytometric (Figure 3A) or western blotting (Figure 3B) analysis. Since COX-2 could promote tumor progression by inducing various effects such as invasion, angiogenesis and suppression of apoptosis as mentioned before, a major function of HER2 in tumorigenesis could be mediated through the up-regulation of COX-2. Since high frequency of R2N1d cells expressed CD44 (Figure 5A) which was found to mediate invasion [14], there could be a synergistic effect of CD44 and COX-2 on inducing the ability of invasion of these cells.

Unlike the parental cell lines developed in hormone/growth factor-enriched medium (M13SV1R2 and M13SV1R2N1) (Additional file 5, Figure S3), the R2d and R2N1d cells developed in hormone/growth factor-deprived medium contain CD44^+^/CD24^- ^cells ([16] and this study). The frequency of these cells was much higher in R2N1d cells (~50%) than in R2d cells (~10%) (Figure 5A). The results indicate that HER2 may sustain tumor growth and promote distant metastasis [43] by increasing the frequency of CD44^+^/CD24^- ^cancer stem cells. It is noted from our previous study [16] that R2d cells were non-tumorigenic. However, after the exposure to estrogen, these cells increased the expression of CD44^+^/CD24^- ^(from 10% to 15%) and became tumorigenic. A previous study reported that Herceptin treatment could decrease ALDH-positive cells in a HER2-expressing breast carcinoma cell line [20]. The side population of breast carcinoma, MCF-7, cell line was tumorigenic and expressed high level of Notch-1 and beta-catenin besides ABCG2, suggesting that side population has some intrinsic properties of stem cells [44]. Recently, HER2 expression was found to increase the frequencies of side population in both luminal and basal subtypes of breast cancers [45]. These observations and the results of our study using different breast cancer stem cell markers provide independent evidence that HER2 has the ability to maintain high frequency of tumor stem cells.

The expression of histone deacetylase, HDAC6, was found to be higher in R2N1d cells than R2d cells (2.41 folds) by microarray study (Additional file 1, Table S1), although both cell lines show high expression by flow cytometric analysis (Figure 3C). This gene can be down-regulated by treatment with HER2 and PI3K inhibitors (Figure 3D). Except for the regulation of cell motility by HDAD6 via estrogen signaling [46], not much is known about the regulation of HDAC6 in human breast cancer. The results of this study provide evidence that HDAC6 expression could be regulated by HER2 and AKT1 in breast cancer. The regulation of Akt phosphorylation by HDAC6 has been reported [36]. Therefore, it is possible that HER2 may activate PI3K/Akt activity through up-regulation of HDAC6. Like COX-2, HDAC6 could induce pleiotropic effects on tumorigenesis and tumor progression [35-39]. It appears that both are key genes that mediate HER2 effects on tumor progression.

### Modulation of HER2 expression by cell culture condition and tumorigenesis

Non-adherent cells derived from CD44^+^/CD24^- ^R2N1d cells were found to lose the expression of HER2, HDAC and AKT simultaneously (Figure 5C, D, E). Apparently, the non-adherent cells are resistant to anoikis by a mechanism in the absence of Akt expression [47]. After replating and reattachment as adherent culture, these cells regained the expression of these 3 genes. We have carried out experiments to test the importance of the expression of these genes in tumor development (Figure 6 and 7). The comparative study showed that non-adherent R2N1d cells formed tumors with HER2 expression but at lower frequency (4/6 vs. 6/6) and with much longer latency period (6 months vs. 2 weeks). The results reaffirm the important role of HER2 in tumor development and show that HER2 expression can be modulated by cell culture condition.

We speculate that HER2 expression could be modulated by changed *in vivo *condition following chemotherapy and/or radiation treatment. The few remaining cancer stem cells under the changed environment may not express HER2 and remain non-tumorigenic for a long time. However, these cells could express HER2 and become tumorigenic in response to tissue or humoral change. This may be a mechanism for tumor dormancy [48] and relapse.

Overall, this study provides clear evidence that HER2 has the ability to induce fast-growing invasive breast tumors of stem cell origin. Considering the key genes induced by HER2 and their biological effects, it appears that the up-regulation of the expression of COX-2 and HDAC6, and the increase in CD44^+^/CD24^- ^cancer stem cell frequency may account for the potent tumorigenic function of HER2 in breast carcinogenesis. To counter these HER2 effects, future therapy of HER2-positive breast tumors may consider a strategy of using the combination of anti-HER2 antibodies with other drugs that target breast cancer stem cells such as metfdormin [49,50], salinomycin [51] and CXCR1 [52] to eliminate breast cancer stem cells.

## Methods

### Development of R2d and R2N1d cells

Previously, we have reported the development of immortal (M13SV1), weakly tumorigenic (M13SV1R2) and highly tumorigenic (M13SV1R2N1) cell lines from a human breast epithelial cell type with stem cell characteristics after successive SV40 large T-antigen transfection, X-ray irradiation and ectopic expression of C-erbB2/neu oncogene [25,28]. These M13SV1R2 cells lost their tumorigenicity concomitant with the expression of two tumor suppressor genes, maspin and alpha-6 integrin, after culturing in a growth factor/hormone-deprived medium for >10 passages (referred to as R2d) [16]. In this study, M13SV1R2N1 cells were cultured in the MSU-1 medium supplemented with growth factors/hormones and 5% fetal bovine serum (FBS) [21]. After one week culture in this medium, M13SV1R2N1 cells were subcultured in the basic MSU-1 medium with 5% FBS without other growth factors/hormones and passaged more than 10 times (referred to as R2N1d cells) (Figure 1A).

### Immunocytochemical analysis of gene expression

For immunostaining, cells were fixed by 4% paraformaldehyde in phosphate buffered saline (PBS). After rinsing with PBS, the cells were permeabilized (0.5% triton X-100) for 10 min. These cells were then incubated with primary antibodies (ant-HER2 or anti-ER-α) at 25℃ overnight. The following day, these cells were incubated with a secondary antibody conjugated with fluorescein isothiocyanate (FITC) (50 μg/ml, Sigma, USA) for 1 hr at 25℃. For nuclear staining, the cells were washed with PBS before incubation with 4', 6 diamidino-2-phenylindole (DAPI, Sigma, USA) (1 μg/ml in PBS) for 5 min.

### Flow cytometric analysis of gene expression

Following trypsinization, cells were strained through a 40 μM nylon mesh to ensure the obtaining of single cells and suspended in ice-cold solution for a density of 1 × 10^6 ^cells/ml. Antibodies (Notch-4, Msi1, Notch-1, CK-18, CK-19, Oct-4, HDAC6, COX2 and HER2; CD24 conjugated with FITC; CD44 conjugated with phycoerythrin, PE) were added to the cell suspension at concentrations suggested by the manufacturer and cells were incubated at 4°C in the dark for 45 min. Then the cells were incubated with a secondary antibody conjugated with FITC or PE for 1 hr at 4°C. These labeled cells were washed twice, suspended in PBS and analyzed using a flow cytometer (FACS Calibur, Becton Dickinson). As negative controls, cells were stained with either isotype-matched control antibodies or with no primary antibody. No difference was observed between these two controls.

### Western blotting

The proteins were extracted with 20% SDS lysis solution containing several protease and phosphatase inhibitors (1 mM phenylmethylsulfonyl fluoride, 1 mM leupeptin, 1 mM antipain, 0.1 mM aprotinin, 0.1 mM sodium orthovanadate, 5 mM sodium fluoride). Protein concentrations were measured using Biorad Protein Quantification kit (Biorad, CA, USA). Equal amounts of protein (15 μg/lane) were separated by 12% SDS-PAGE and transferred from the gel to PVDF membranes (Millipore Corp, Bedford, MA). Immunoblotting was carried out using monoclonal antibody (anti-COX-2, anti-COX-1, anti-AKT1, anti-HER2, anti-Oct4, anti-HDAC6, anti-alpha-tubulin and anti-β-Actin). This was then followed by incubation with horseradish peroxidase-conjugated secondary antibody and detected with the ECL chemiluminescent detection reagent (Amersham Co., IL, USA). The membranes were exposed to X-ray film for 15 s to 3 min.

### Reverse transcription-polymerase chain reaction (RT-PCR)

5 μg of total RNA extracted from cells were used to synthesize the first-strand cDNA, using the Reverse Transcription System (Promega, A3500) according to the manufacturer's protocol. PCR amplification was carried out by using 1 μL of the first-strand cDNA as a template in a total volume of 15 μL containing 1 μL of each primer (10 pmol/L) and 7.5 μL of EconoTaq ^® ^PLUS GREEN Master Mix Kit (Lucigen, F93481-1). The primers used are as listed for *HER2 *(forward, 5'-CCCGAAACGTGCTAGTCAAGAG-3'; reverse, 5'-TGCAGATTGGAGGCTGAG GTAG-3') and *HDAC6 *(forward, 5'-CCAGCTAACCCACCTGCTCATG-3'; reverse, 5'-GGGCTTCCAGAGCACAGGAAAC-3'). Following 1 minute denaturation at 95°C, the reactions were cycled 30 times with 45 seconds denaturation at 95°C and 30 seconds annealing at 55°C and then extension at 72°C for 1 min. The reactions were performed in the DNA Thermal Cycler 480 (Takara). The last polymerization step was at 72°C for 10 min.

### Invasion assay

Cells were inoculated into 24-well Matrigel™ Invasion inserts (2.5 × 10^5 ^cells/well) (8 μm pore size) (BD Biosciences, USA). Inserts were placed into Falcon companion plates and incubated for 24 hr for invasion. Following incubation, media plus cells were removed from the top chamber using cotton swabs and PBS. The number of cells invading to the underside of the membrane was determined. The data are presented as the average number of invading cells per well in triplicate.

### Tumorigenicity in SCID mice

Female immune-deficient (SCID) mice (BALB/cAnN.Cg-*Foxn1*^nu^/CrlNarl, 4 to 6-week-old) were obtained from the National Laboratory Animal Center (Taipei, Taiwan). Different numbers of R2d, R2N1d, non-adherent R2N1d and re-attached R2N1d cells (1 × 10^5^, n = 6; 1 × 10^6^, n = 6; 1 × 10^7^, n = 6), were inoculated subcutaneously into female immune-deficient (SCID) mice, 2 sites for each mouse. Tumors developed were dissected, measured and histologically examined.

### Immunohistochemical study of gene expression in tumor tissues

Serially cut tumor sections (4 um thick) were processed and incubated with primary antibodies against Ki-67 (1:75), COX-2 (1:50) (Dako, Denmark), matrix metalloproteinase-9 (MMP-9) (1:75), (Neomarkers, USA), HER2 (Dako, Denmark) (1:75) and vascular endothelial growth factor (VEGF) (1:150) (Santa Cruz Biotechnology, USA) at room temperature for 1 hr. The sections were then incubated in 3, 3-diaminobenzidine solution for 5 min, followed by Mayer's haematoxylin counterstaining and mounting. Negative controls were treated with non-immune serum instead of primary antibody.

The classification and evaluation of the expression of pathological markers in tumor tissues were as described [16].

### Statistical analysis

Results shown are representative of at least three separate experiments. The significance of difference between treatments was assessed by the Mann-Whitney test of nonparametric statistics and was carried out using SPSS for Windows 13.0 statistics program (SPSS Inc., Chicago, USA). The p value < 0.05 was considered to be significant. All statistical data are presented as mean ± SD.

## Competing interests

The authors declare that they have no competing interests.

## Authors' contributions

KHW and APK, conception, experimental design and performance, data analysis and interpretation, manuscript writing; CCC conception and design, data analysis and interpretation; JNL, MFH, CYL and HSC performed research; EMT conception and design, financial support, provision of study material or patients, final approval of manuscript. All the authors read and approved the final manuscript.

## Supplementary Material

Additional file 1 Table S1: The effects of HER2 on gene expression in R2N1d cells as indicated by R2N1d/R2d ratio Click here for file

Additional file 2 Figure S1: The HER2 effect on up-regulation of COX2 expression is confirmed by western blot analysis when R2N1d and R2d cells are compared. Click here for file

Additional file 3 Figure S2: The expression of CD44^+^/CD24^- ^in non-adherent R2N1d cells was found to be dramatically reduced compared to adherent cells. Click here for file

Additional file 4 **Table S2: Characteristics of M13SV1R2, R2d, M13SV1R2N1 and R2N1d cell line**. Click here for file

Additional file 5 Figure S3: The parental cell lines (M13SV1R2 and M13SV1R2N1) developed in hormone/growth factor-enriched medium. Click here for file
