# Supplementary material for: Increasing CD44+/CD24- tumor stem cells, and upregulation of COX-2 and HDAC6, as major functions of HER2 in breast tumorigenesis
Source: Mol Cancer. 2010 Nov 2;9:288. doi: 10.1186/1476-4598-9-288 (PMC2989327; doi:10.1186/1476-4598-9-288)
Supplement: Additional file 1 — Table S1: The effects of HER2 on gene expression in R2N1d cells as indicated by R2N1d/R2d ratio [file 1476-4598-9-288-S1.DOC]

# Additional file 1

## Table S1 - The effects of HER2 on gene expression in R2N1d cells as indicated by R2N1d/R2d ratio

| # | Gene Symbol | Signal (R2N1d/R2d) | Adhesion | Migration | Metastasis | Inflammation | Agiogenesis |
| --- | --- | --- | --- | --- | --- | --- | --- |
| 1 | SERPINA3 | 48.66 | - | - | V | V | - |
| 2 | HSPA1B | 42.22 | - | - | - | V | - |
| 3 | MMP9 | 33.60 | - | - | V | V | - |
| 4 | S100A9 | 32.18 | - | - | - | V | - |
| 5 | SRPX | 21.63 | V | - | - | - | - |
| 6 | TNFRSF6B | 21.39 | - | - | V | - | - |
| 7 | TIMP1 | 20.26 | - | - | V | V | - |
| 8 | UPP1 | 16.93 | - | - | V | - | - |
| 9 | EGR1 | 16.67 | - | - | V | - | - |
| 10 | HSPD1 | 15.54 | - | - | V | V | - |
| 11 | DCBLD1 | 15.30 | V | - | - | - | - |
| 12 | MT2A | 15.28 | - | - | - | V | - |
| 13 | SLC20A1 | 14.49 | - | - | - | V | - |
| 14 | LAMC2 | 14.28 | V | - | V | - | - |
| 15 | TNFRSF12A | 14.09 | V | - | V | V | V |
| 16 | BTG1 | 13.71 | - | V | - | - | - |
| 17 | VIM | 13.39 | - | - | V | - | - |
| 18 | METAP2 | 12.99 | - | - | V | - | - |
| 19 | LCN2 | 12.87 | - | - | V | V | - |
| 20 | HSPA9 | 12.47 | - | - | V | - | - |
| 21 | HBEGF | 12.38 | - | - | - | - | V |
| 22 | PRSS3 | 12.19 | - | - | - | V | - |
| 23 | S100A11 | 12.12 | - | - | V | V | - |
| 24 | APEX1 | 11.61 | - | - | V | - | - |
| 25 | TNFRSF11B | 11.42 | - | - | V | V | - |
| 26 | CTNNA1 | 11.03 | V | - | V | - | - |
| 27 | COL6A3 | 11.00 | V | - | - | - | - |
| 28 | SAT1 | 10.90 | - | - | - | V | - |
| 29 | DAP3 | 10.81 | - | - | - | V | - |
| 30 | LOX | 10.38 | - | - | V | - | - |
| 31 | LAMA4 | 10.21 | V | - | V | - | - |
| 32 | LAMA3 | 10.21 | V | - | - | - | - |
| 33 | CD97 | 10.15 | V | - | - | - | - |
| 34 | HSPA4 | 10.04 | - | - | V | V | - |
| 35 | PLAU | 10.01 | - | - | V | V | V |

Table 1 (continue)

| # | Gene Symbol | Signal (R2N1d/R2d) | Adhesion | Migration | Metastasis | Inflammation | Agiogenesis |
| --- | --- | --- | --- | --- | --- | --- | --- |
| 36 | HNRNPK | 9.99 | - | - | V | - | - |
| 37 | ARID4A | 9.95 | - | - | V | - | - |
| 38 | PTPRF | 9.85 | V | - | - | - | - |
| 39 | HMGA1 | 9.81 | - | - | V | - | - |
| 40 | CD44 | 9.79 | V | V | V | V | - |
| 41 | IL1A | 9.76 | - | - | V | - | - |
| 42 | HIF1A | 9.63 | - | - | V | V | V |
| 43 | IL8 | 9.60 | - | - | V | V | V |
| 44 | DKK1 | 9.54 | - | - | V | - | - |
| 45 | HEXB | 9.54 | - | - | - | V | - |
| 46 | KLK8 | 9.50 | - | - | V | - | - |
| 47 | NFE2L2 | 9.50 | - | - | - | V | - |
| 48 | NFKB1 | 9.39 | - | - | V | V | - |
| 49 | IDH1 | 9.38 | - | - | V | - | - |
| 50 | PTTG1 | 9.37 | - | - | V | - | - |
| 51 | TGFBI | 9.32 | V | - | V | - | - |
| 52 | C1QBP | 9.21 | - | - | - | V | - |
| 53 | OCIAD1 | 9.02 | - | - | V | - | - |
| 54 | DCBLD2 | 9.00 | V | - | - | - | - |
| 55 | ALCAM | 8.98 | V | - | V | - | - |
| 56 | HSP90B1 | 8.73 | - | - | V | - | - |
| 57 | HSPA5 | 8.68 | - | - | V | - | - |
| 58 | PTGDR | 8.64 | - | - | - | V | - |
| 59 | ANXA1 | 8.60 | - | - | V | - | - |
| 60 | HMOX1 | 8.60 | - | - | V | V | V |
| 61 | NDRG1 | 8.49 | - | - | V | - | - |
| 62 | ENO1 | 8.44 | - | - | V | - | - |
| 63 | RHOA | 8.43 | V | - | V | - | - |
| 64 | ITGA5 | 8.39 | V | - | - | V | - |
| 65 | MYC | 8.37 | - | - | V | - | - |
| 66 | ACTR2 | 8.35 | - | - | V | - | - |
| 67 | CTNNAL1 | 8.32 | V | - | - | - | - |
| 68 | BAG3 | 8.31 | - | - | V | - | - |
| 69 | CREB1 | 8.15 | - | - | - | V | - |
| 70 | NCK1 | 8.12 | - | V | - | - | - |

Table 1 (continue)

| # | Gene Symbol | Signal (R2N1d/R2d) | Adhesion | Migration | Metastasis | Inflammation | Agiogenesis |
| --- | --- | --- | --- | --- | --- | --- | --- |
| 71 | NUP62 | 8.11 | - | V | - | - | - |
| 72 | CASK | 8.05 | V | - | - | - | - |
| 73 | NT5E | 8.04 | - | - | V | - | - |
| 74 | FXYD5 | 8.03 | - | - | V | - | - |
| 75 | HMMR | 7.99 | - | - | V | - | - |
| 76 | HSPE1 | 7.97 | - | - | V | - | - |
| 77 | RRM1 | 7.96 | - | - | V | - | - |
| 78 | ANXA2 | 7.96 | - | - | - | - | V |
| 79 | CDK4 | 7.92 | - | - | V | - | - |
| 80 | SHC1 | 7.69 | - | - | - | - | V |
| 81 | BIRC2 | 7.69 | - | - | V | V | - |
| 82 | BIRC3 | 7.69 | - | - | - | V | - |
| 83 | SMYD3 | 7.68 | - | - | V | - | - |
| 84 | PNN | 7.61 | V | - | - | - | - |
| 85 | SPOCK1 | 7.55 | V | - | - | - | - |
| 86 | ADM | 7.52 | - | - | V | V | - |
| 87 | TNFRSF10A | 7.51 | - | - | V | - | - |
| 88 | TNFRSF10B | 7.51 | - | - | - | V | - |
| 89 | KIF2C | 7.48 | - | - | V | - | - |
| 90 | ITGA2 | 7.47 | V | - | - | - | - |
| 91 | EZR | 7.42 | - | - | V | - | - |
| 92 | ABCF1 | 7.40 | - | - | - | V | - |
| 93 | FRAP1 | 7.35 | - | - | - | V | - |
| 94 | ODC1 | 7.32 | - | - | V | - | - |
| 95 | C14orf166 | 7.31 | - | - | V | - | - |
| 96 | LEPR | 7.31 | - | - | V | - | V |
| 97 | ASAP1 | 7.23 | - | - | V | - | - |
| 98 | VEGFC | 7.16 | - | - | V | V | V |
| 99 | CCNA2 | 7.13 | - | - | V | - | - |
| 100 | PSMD1 | 7.12 | - | - | - | V | - |
| 101 | SMAD3 | 7.11 | - | - | - | V | - |
| 102 | ADIPOR1 | 7.09 | - | - | - | V | - |
| 103 | STC1 | 7.08 | - | - | V | V | - |
| 104 | EREG | 7.07 | - | - | V | - | V |
| 105 | MAP3K7 | 7.07 | - | - | V | - | V |

Table 1 (continue)

| # | Gene Symbol | Signal (R2N1d/R2d) | Adhesion | Migration | Metastasis | Inflammation | Agiogenesis |
| --- | --- | --- | --- | --- | --- | --- | --- |
| 106 | IGF2BP3 | 7.02 | - | - | V | - | - |
| 107 | ITGB1BP1 | 6.99 | V | V | - | - | - |
| 108 | KIT | 6.95 | - | - | V | - | - |
| 109 | FOS | 6.94 | - | - | V | V | - |
| 110 | KLF6 | 6.91 | - | - | V | - | - |
| 111 | TMBIM4 | 6.89 | - | - | V | - | - |
| 112 | CTSL1 | 6.87 | - | - | V | - | - |
| 113 | THBS2 | 6.87 | V | - | - | - | - |
| 114 | NAMPT | 6.86 | - | - | - | V | - |
| 115 | CEACAM1 | 6.85 | - | V | V | V | V |
| 116 | F2RL1 | 6.84 | - | - | - | V | - |
| 117 | SLC3A2 | 6.84 | - | - | V | - | - |
| 118 | ITGAV | 6.84 | V | - | V | - | V |
| 119 | KLK1 | 6.74 | - | - | - | V | - |
| 120 | KLK6 | 6.74 | - | - | V | - | - |
| 121 | KLK7 | 6.74 | - | - | V | - | - |
| 122 | AATF | 6.68 | V | - | - | - | - |
| 123 | IL6 | 6.68 | - | - | - | V | - |
| 124 | ANXA7 | 6.67 | - | - | V | - | - |
| 125 | SDCBP | 6.66 | - | - | V | - | - |
| 126 | TRIM32 | 6.66 | - | - | V | - | - |
| 127 | SERPINB5 | 6.65 | - | - | V | - | - |
| 128 | SPAG9 | 6.63 | - | - | V | - | - |
| 129 | CFH | 6.58 | - | - | - | V | - |
| 130 | SRGAP1 | 6.58 | - | V | - | - | - |
| 131 | GPR56 | 6.56 | V | - | V | - | - |
| 132 | TSPAN5 | 6.55 | V | - | - | - | - |
| 133 | NCOA4 | 6.55 | - | - | V | V | - |
| 134 | LLGL1 | 6.51 | - | - | V | - | - |
| 135 | NRP1 | 6.47 | V | V | - | - | V |
| 136 | NELL1 | 6.47 | V | - | - | - | - |
| 137 | TGFBR2 | 6.44 | - | - | V | - | - |
| 138 | SLC2A3 | 6.41 | - | - | V | - | - |
| 139 | MCM2 | 6.41 | - | - | V | - | - |
| 140 | HMGB1 | 6.40 | - | - | V | V | - |

Table 1 (continue)

| # | Gene Symbol | Signal (R2N1d/R2d) | Adhesion | Migration | Metastasis | Inflammation | Agiogenesis |
| --- | --- | --- | --- | --- | --- | --- | --- |
| 141 | EPCAM | 6.38 | - | - | V | - | - |
| 142 | SLC2A1 | 6.38 | - | - | V | - | - |
| 143 | PTPRK | 6.35 | V | - | - | - | - |
| 144 | CXADR | 6.35 | V | - | - | - | - |
| 145 | ACO1 | 6.33 | - | - | - | V | - |
| 146 | SFN | 6.31 | - | - | V | - | - |
| 147 | SIRT1 | 6.30 | - | - | - | V | - |
| 148 | LGALS3 | 6.29 | - | - | V | - | - |
| 149 | TMBIM6 | 6.29 | - | - | V | - | - |
| 150 | NARG1 | 6.29 | - | - | - | - | V |
| 151 | FKBP4 | 6.26 | - | - | - | V | - |
| 152 | WISP2 | 6.26 | V | - | V | - | - |
| 153 | HMGCR | 6.23 | - | - | - | V | - |
| 154 | DDR2 | 6.14 | V | - | - | - | - |
| 155 | CIB1 | 6.14 | V | - | - | - | - |
| 156 | RND3 | 6.09 | V | - | - | - | - |
| 157 | TJP1 | 6.08 | - | - | V | - | - |
| 158 | CCL13 | 6.07 | - | - | - | V | - |
| 159 | TNS3 | 6.07 | - | V | - | - | - |
| 160 | CCL5 | 6.07 | V | - | - | V | - |
| 161 | TUSC3 | 6.02 | - | - | V | - | - |
| 162 | FOSB | 6.01 | - | - | - | V | - |
| 163 | MGST1 | 6.01 | - | - | - | V | - |
| 164 | ADIPOR2 | 5.98 | - | - | - | V | - |
| 165 | ELF3 | 5.98 | - | - | - | V | - |
| 166 | LAMC1 | 5.97 | V | V | V | - | - |
| 167 | CSNK1A1 | 5.92 | - | - | V | - | - |
| 168 | HSP90AA1 | 5.86 | - | - | - | V | - |
| 169 | F11R | 5.86 | V | - | V | V | - |
| 170 | VEZF1 | 5.85 | - | - | - | - | V |
| 171 | PCNA | 5.82 | - | - | V | - | - |
| 172 | HDAC1 | 5.82 | - | - | V | - | - |
| 173 | PCDHB16 | 5.80 | V | - | - | - | - |
| 174 | NRD1 | 5.78 | - | V | - | - | - |
| 175 | ATP5B | 5.77 | - | - | - | - | V |

Table 1 (continue)

| # | Gene Symbol | Signal (R2N1d/R2d) | Adhesion | Migration | Metastasis | Inflammation | Agiogenesis |
| --- | --- | --- | --- | --- | --- | --- | --- |
| 176 | PMEPA1 | 5.75 | - | - | V | - | - |
| 177 | CDH15 | 5.73 | V | - | - | - | - |
| 178 | CDH3 | 5.73 | V | - | - | - | - |
| 179 | RALGDS | 5.71 | - | - | V | - | - |
| 180 | FYN | 5.70 | - | - | V | - | - |
| 181 | TNFRSF21 | 5.70 | - | - | - | V | - |
| 182 | GDI2 | 5.63 | - | - | V | - | - |
| 183 | CXCL16 | 5.63 | - | - | - | V | - |
| 184 | PPT1 | 5.62 | - | - | V | - | - |
| 185 | SCARB2 | 5.60 | V | - | - | - | - |
| 186 | EPAS1 | 5.60 | - | - | - | - | V |
| 187 | CCNB1 | 5.59 | - | - | V | - | - |
| 188 | LPXN | 5.59 | V | - | - | - | - |
| 189 | COL6A1 | 5.58 | V | - | - | - | - |
| 190 | TNFRSF1A | 5.58 | - | - | - | V | - |
| 191 | PTGES | 5.57 | - | - | - | V | - |
| 192 | GJB2 | 5.56 | - | - | V | - | - |
| 193 | CDH1 | 5.56 | V | - | V | V | - |
| 194 | TXN | 5.56 | - | - | V | V | - |
| 195 | TNFSF13B | 5.54 | - | - | - | V | - |
| 196 | GHITM | 5.53 | - | - | V | - | - |
| 197 | TYMS | 5.52 | - | - | V | - | - |
| 198 | MTSS1 | 5.51 | V | - | V | - | - |
| 199 | MTA3 | 5.51 | - | - | V | - | - |
| 200 | ID1 | 5.49 | - | - | V | - | - |
| 201 | SIP1 | 5.49 | - | - | V | - | - |
| 202 | TMEM49 | 5.49 | - | - | V | - | - |
| 203 | IL1B | 5.49 | - | - | V | V | V |
| 204 | RAN | 5.47 | - | - | V | - | - |
| 205 | HES5 | 5.41 | V | - | - | - | - |
| 206 | MGMT | 5.39 | - | - | V | - | - |
| 207 | CX3CL1 | 5.36 | V | - | - | V | - |
| 208 | LAMP1 | 5.34 | - | - | V | - | - |
| 209 | CLDN7 | 5.33 | - | - | V | - | - |
| 210 | SSR4 | 5.32 | - | - | V | - | - |

Table 1 (continue)

| # | Gene Symbol | Signal (R2N1d/R2d) | Adhesion | Migration | Metastasis | Inflammation | Agiogenesis |
| --- | --- | --- | --- | --- | --- | --- | --- |
| 211 | PARP1 | 5.30 | - | - | V | V | - |
| 212 | SOX18 | 5.29 | - | - | - | - | V |
| 213 | G3BP1 | 5.28 | - | - | V | - | - |
| 214 | IKBKG | 5.27 | - | - | - | V | - |
| 215 | PROC | 5.27 | - | - | - | V | - |
| 216 | UCHL1 | 5.24 | - | - | V | V | - |
| 217 | SERPINE1 | 5.24 | - | - | V | V | - |
| 218 | TNC | 5.21 | V | - | V | - | - |
| 219 | CTHRC1 | 5.16 | - | V | V | - | - |
| 220 | NSMAF | 5.14 | - | - | - | V | - |
| 221 | TNFSF14 | 5.09 | - | - | V | V | - |
| 222 | NCOA6 | 5.07 | - | - | - | V | - |
| 223 | MTDH | 5.07 | - | - | V | - | - |
| 224 | LPP | 5.05 | V | - | - | - | - |
| 225 | CDC2 | 5.04 | - | - | V | - | - |
| 226 | ANXA5 | 5.04 | - | - | - | V | - |
| 227 | RAD21 | 5.04 | - | - | V | - | - |
| 228 | SEMA4D | 5.02 | V | - | - | - | - |
| 229 | PRDX6 | 5.01 | - | - | - | V | - |
| 230 | HDAC6 | 2.41 | - | V | V | - | - |
|  |  | Total genes | 51 | 13 | 136 | 79 | 21 |
