# Supplementary figures and images for: Increasing CD44+/CD24- tumor stem cells, and upregulation of COX-2 and HDAC6, as major functions of HER2 in breast tumorigenesis
Source: Mol Cancer. 2010 Nov 2;9:288. doi: 10.1186/1476-4598-9-288 (PMC2989327; doi:10.1186/1476-4598-9-288)

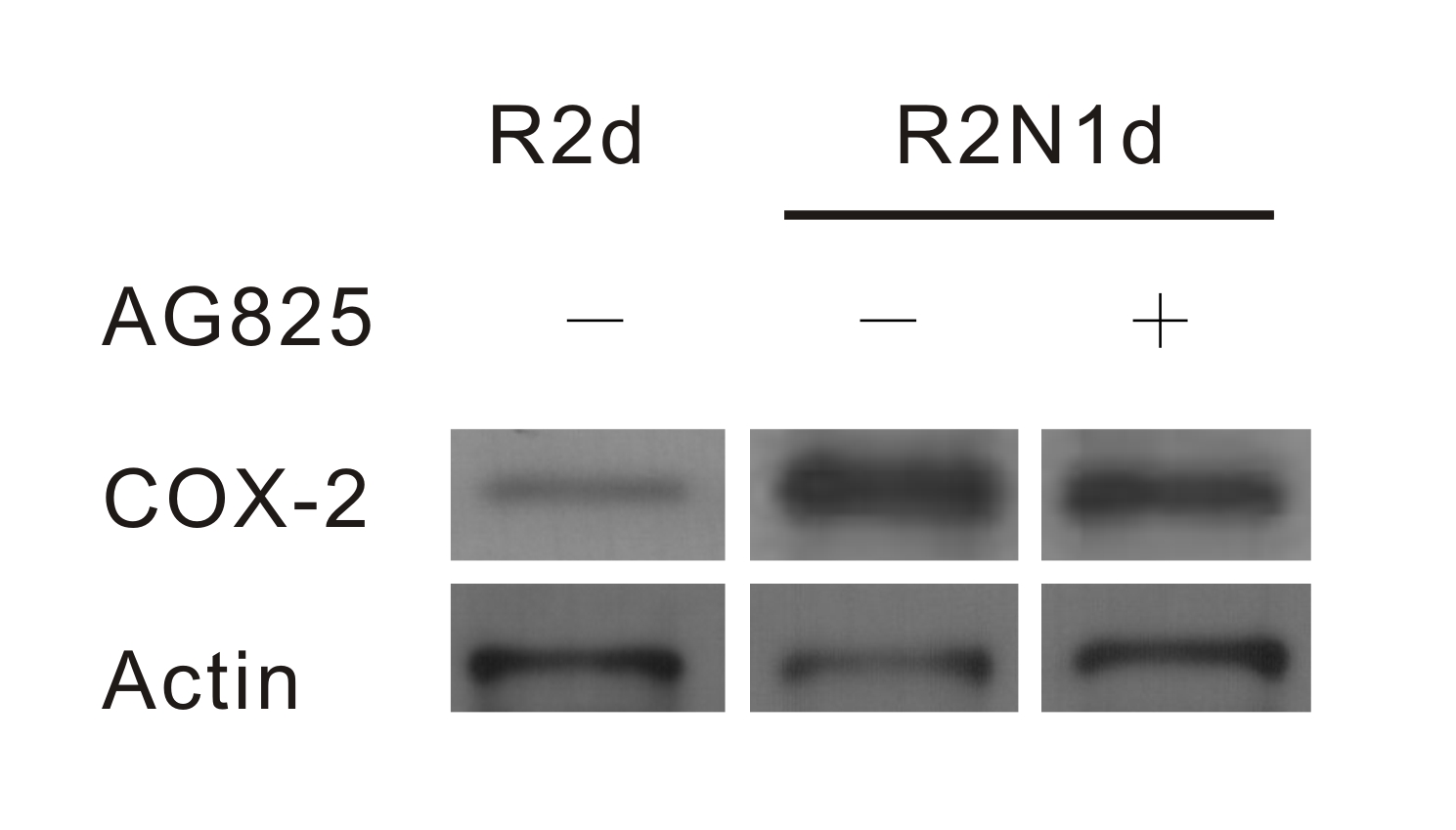

Supplement: Additional file 2 — Figure S1: The HER2 effect on up-regulation of COX2 expression is confirmed by western blot analysis when R2N1d and R2d cells are compared. [file 1476-4598-9-288-S2.JPEG]

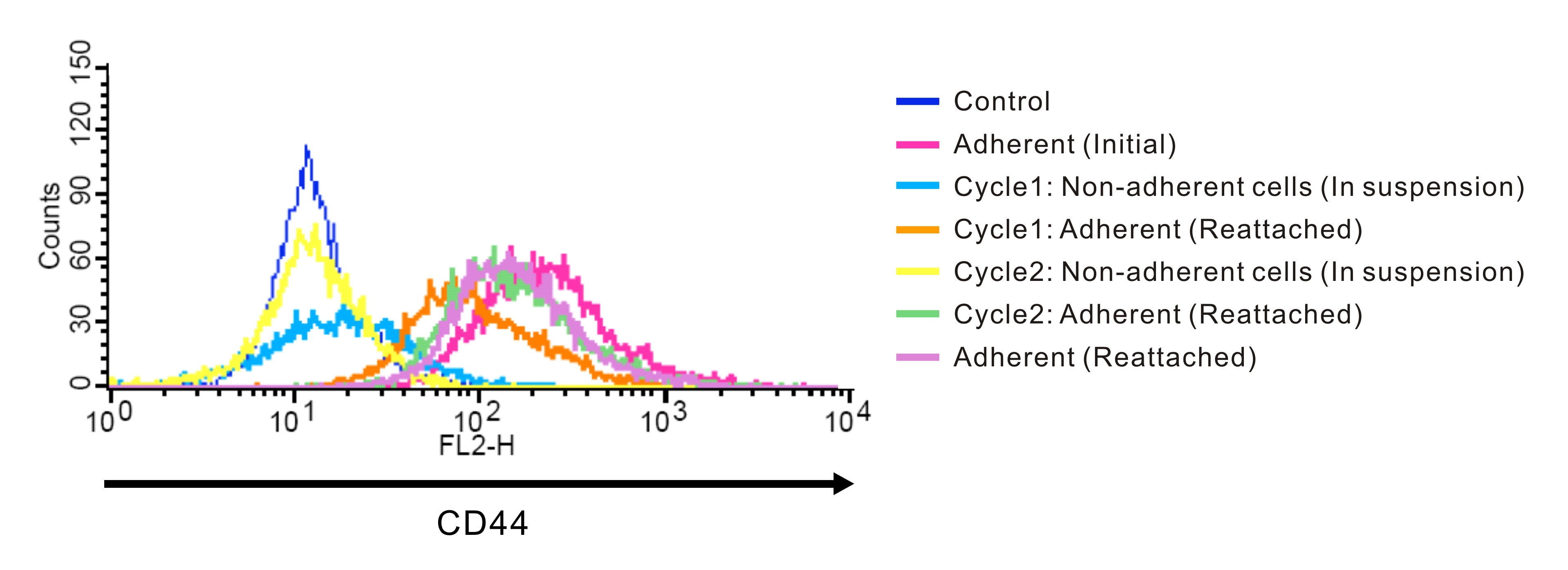

Supplement: Additional file 3 — Figure S2: The expression of CD44+/CD24- in non-adherent R2N1d cells was found to be dramatically reduced compared to adherent cells. [file 1476-4598-9-288-S3.JPEG]

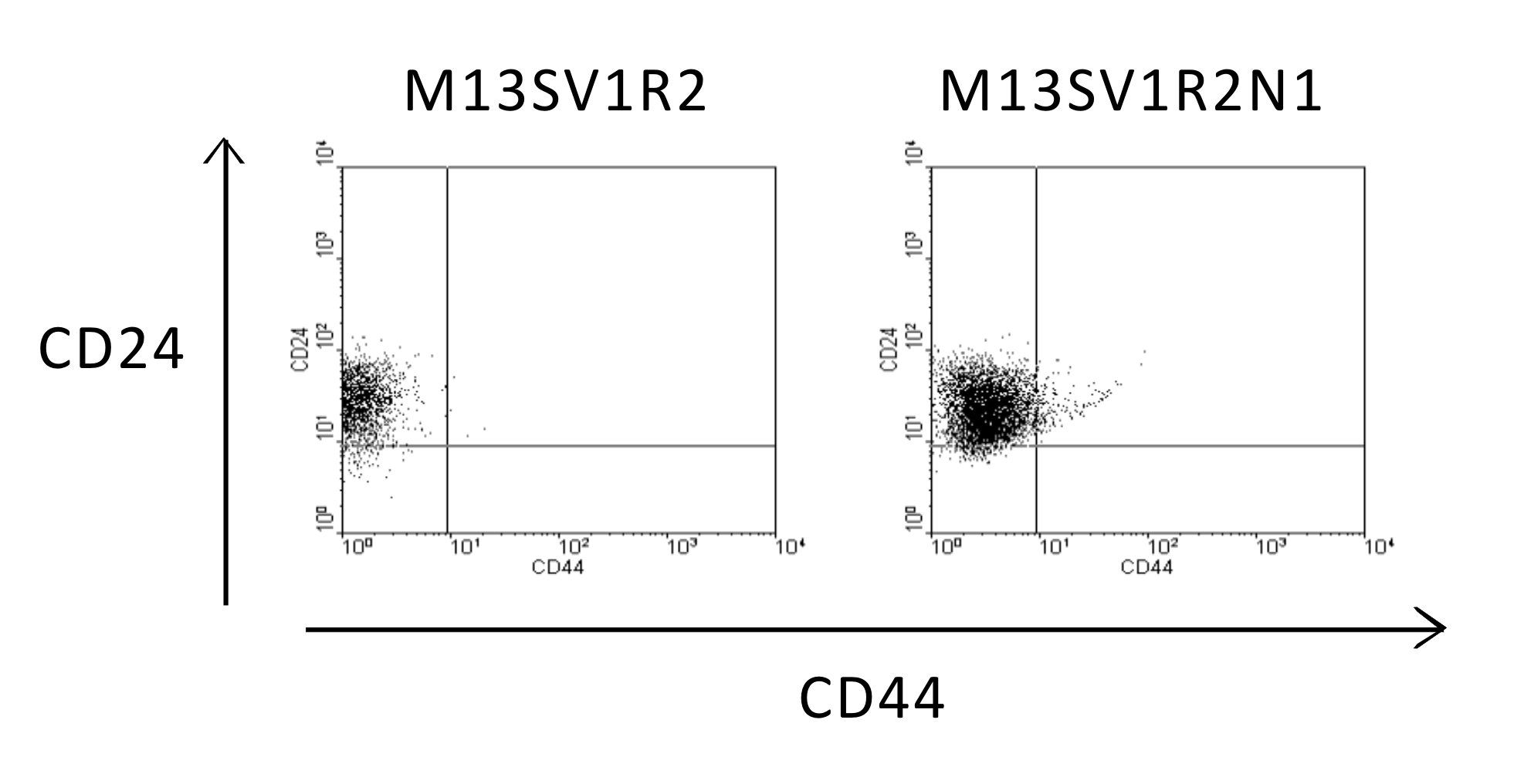

Supplement: Additional file 5 — Figure S3: The parental cell lines (M13SV1R2 and M13SV1R2N1) developed in hormone/growth factor-enriched medium. [file 1476-4598-9-288-S5.JPEG]
