# Supplementary material for: Increasing CD44+/CD24- tumor stem cells, and upregulation of COX-2 and HDAC6, as major functions of HER2 in breast tumorigenesis
Source: Mol Cancer. 2010 Nov 2;9:288. doi: 10.1186/1476-4598-9-288 (PMC2989327; doi:10.1186/1476-4598-9-288)
Supplement: Additional file 4 — Table S2: Characteristics of M13SV1R2, R2d, M13SV1R2N1 and R2N1d cell line. [file 1476-4598-9-288-S4.DOC]

**Additional file 4**

| **Table S2** - Characteristics of M13SV1R2, R2d, M13SV1R2N1 and R2N1d cell line | | | | |
| --- | --- | --- | --- | --- |
|  | M13SV1R2 | R2d | M13SV1R2N1 | R2N1d |
| Estrogen receptor-alpha | + | + | + | + |
| Stem cell marker |  |  |  |  |
| Oct-4 | + | + | + | + |
| Epithelial surface marker |  |  |  |  |
| Cytokeratin 14 | - | - | - | - |
| Cytokeratin 18 | + | + | + | + |
| Cytokeratin 19 | + | + | + | + |
| Epithelial membrane antigen (EMA) | + | + | + | + |
| E-cadherin | + | + | + | + |
| Tumor suppressor gene |  |  |  |  |
| Maspin | - | + | - | - |
| alpha-6 Integrin | - | + | - | - |
| Tumorigenicity | + | - | + | + |

**Derivation of human breast epithelial cell lines used in this study:**

SV40 X-rays C-erbB2/neu

Type 1 HME13 ==========> M13SV1 ==========> M13SV1R2 ==========> M13SV1R2N1

(normal stem cells) (immortal) (weakly tumorigenic) (highly tumorigenic)

All the above cell lines are cultured in the growth factors/hormone supplemented MSU-1 medium; M13SV1R2 and M13SV1R2N1 cells cultured in growth factors/hormone deprived MSU-1 medium for > 10 passages are designated as R2d and R2N1d cells, respectively (reference 16).
